# Exposure to Common Geographic COVID-19 Prevalence Maps and Public Knowledge, Risk Perceptions, and Behavioral Intentions

**DOI:** 10.1001/jamanetworkopen.2020.33538

**Published:** 2021-01-06

**Authors:** Alistair Thorpe, Aaron M. Scherer, Paul K. J. Han, Nicole Burpo, Victoria Shaffer, Laura Scherer, Angela Fagerlin

**Affiliations:** 1 Department of Population Health Sciences, School of Medicine, University of Utah, Salt Lake City; 2 University of Iowa, Iowa City; 3 Center for Outcomes Research, Maine Medical Center Research Institute, Portland; 4 University of North Carolina, Chapel Hill; 5 University of Missouri, Columbia; 6 University of Colorado, Aurora; 7 Salt Lake City VA Informatics Decision-Enhancement and Analytic Sciences Center for Innovation, Salt Lake City, Utah

## Abstract

This survey study examines knowledge, risk perceptions, and behavioral intentions among survey respondents exposed to different types of COVID-19 prevalence maps.

## Introduction

Several organizations have produced maps showing the prevalence of confirmed coronavirus disease 2019 (COVID-19) cases across the United States, but there is limited data on what map features are most effective at informing the public about infectious disease risk and motivating engagement with recommended health behaviors.^1^ We assessed the association of 6 different COVID-19 maps with knowledge, risk perceptions, and behavioral intentions.

## Methods

This survey study included US adults recruited between May 18 and 28, 2020, by Qualtrics Online Panels. This study was deemed exempt by the University of Iowa institutional review board, given the minimal risk to participants and collection of deidentified information. All respondents provided informed consent and were compensated for their participation. The survey was conducted online in English. This study follows the American Association for Public Opinion Research (AAPOR) reporting guideline.

After providing informed consent, respondents were randomized to see 1 of 6 maps (Figure) or to not receive any information (no map) using an automated function within the Qualtrics software.

**Figure.**
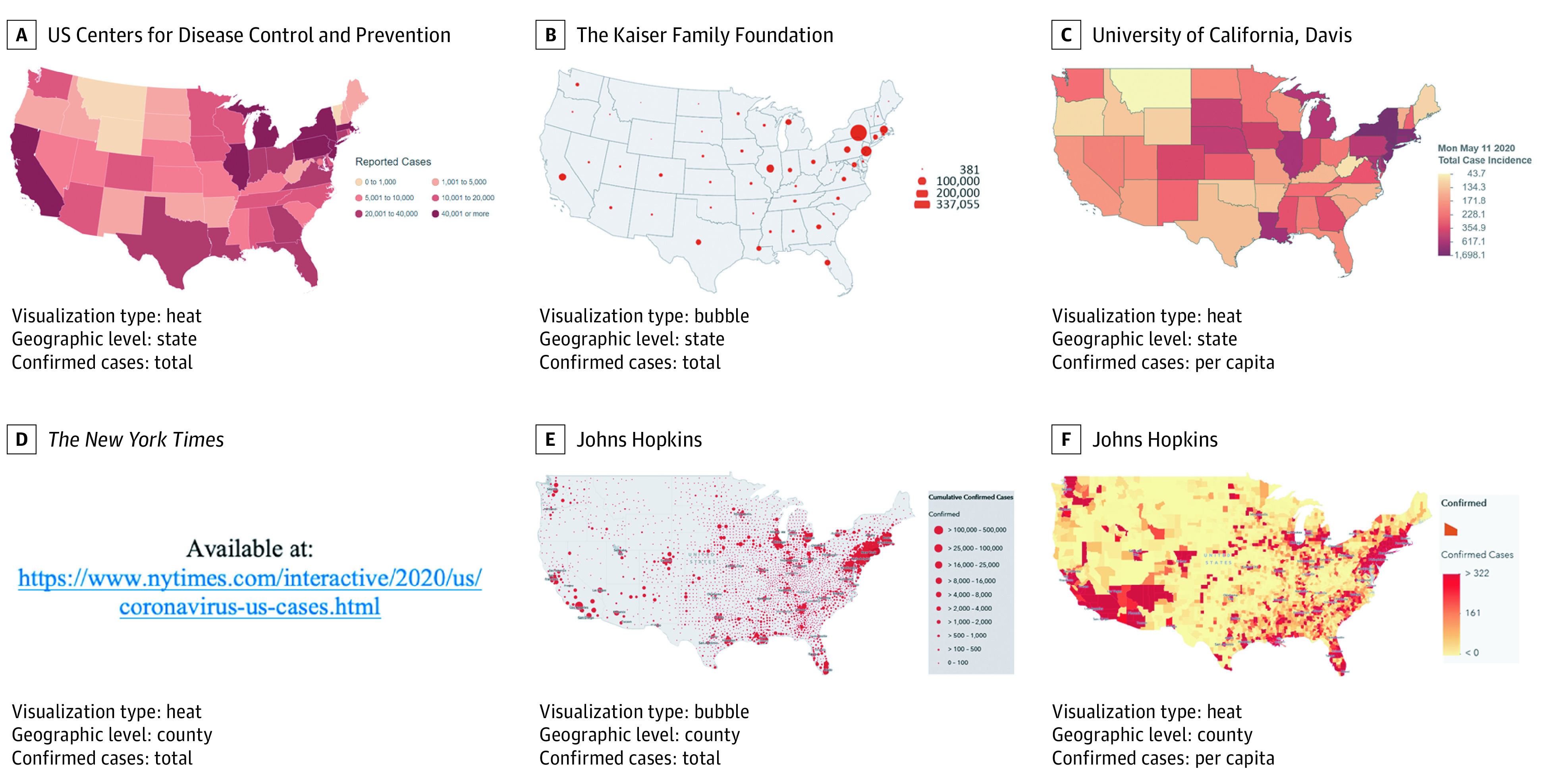
Coronavirus Disease 2019 Maps From Different Publicly Available Sources Maps were collected on May 11, 2020. The *New York Times* map (D) is not included owing to licensing restrictions. The map used in the study was a heat map with a color scheme from light orange for areas with fewer cases moving to red for areas with more cases and gray representing areas with no cases reported. The map showed cases per capita (total reported cases per 100 000 people) at county level and is available on their website (https://www.nytimes.com/interactive/2020/us/coronavirus-us-cases.html). All other maps shown here are also available online: The Centers for Disease Control and Prevention https://www.cdc.gov/coronavirus/2019-ncov/cases-updates/cases-in-us.html (A), The Kaiser Family Foundation (https://www.kff.org/health-costs/issue-brief/state-data-and-policy-actions-to-address-coronavirus/ (B), University of California, Davis (https://covid19.calsurv.org/ (C), and Johns Hopkins (https://coronavirus.jhu.edu/map.html (E and F).

Respondents answered questions assessing their knowledge of confirmed cases of COVID-19 across the US (total cases and cases per capita), their perceived risk of COVID-19 (individual and societal), and their intentions to adhere to infection control guidelines.^2^ Total cases and cases per capita knowledge were each assessed on scales of 4 items specifically about the total or per capita confirmed cases. Scores ranged from 0 to 1, with higher scores indicating greater knowledge about total or per capita numbers of confirmed COVID-19 cases. Individual risk perception was assessed on a scale of 9 items about perceived susceptibility and severity of getting COVID-19. Scores ranged from 1 to 7, with higher scores indicating greater perceived susceptibility and severity of getting COVID-19. Societal risk perception was assessed on a single item about whether the pandemic would be better or worse in 2 weeks. Scores ranged from 1, (indicating that the COVID-19 pandemic would be much worse in 2 weeks) to 7 (indicating the COVID-19 pandemic would be much better in 2 weeks). Intentions to adhere to COVID-19 guidelines were assessed on a scale of 15 guidelines (eg, “avoid gatherings of >10 people”). Scores ranged from 0 to 100, with higher scores indicating greater intent to adhere to the guidelines. Maps were available alongside questions for reference. Using planned contrasts, we compared these outcomes at 4 levels: map intervention (no map vs maps), visualization type (heat vs bubble), geographic level (state vs county), and case format (total vs per capita). Respondents self-reported demographic information, including age, gender, and race/ethnicity.

All tests were 2-sided with *P* values adjusted using Holm-Bonferroni^3^ correction for multiple comparisons. Significance was set at α = .05. Analyses were performed using R Studio statistical software version 1.1.463 (R Project for Statistical Computing).

## Results

After excluding 2062 respondents who did not complete the survey, completed the survey in an unrealistically short time (ie, <9 minutes), or indicated that they did not provide high-quality answers (ie, respondents who answered “I will not provide my best answers” or “I can’t promise either way” to the question “Do you commit to thoughtfully provide your best answers to each question in this survey?”), our final sample included 2676 respondents (completion rate, 57%).

In the final sample, the mean (SD) age was 46 (17) years (range, 18-91 years); 1575 respondents (59%) were women, while 933 respondents (35%) were men, 28 respondents (1%) were transgender or another gender identity, and 140 respondents (5%) did not answer this question. A total of 1663 respondents (62%) were non-Hispanic White, 464 respondents (17%) were Hispanic, 315 respondents (12%) were non-Hispanic Black, 153 respondents (6%) were Asian or Asian American, 34 respondents (1%) were another race, and less than 1% of respondents were American Indian/Alaskan Native or Native Hawaiian/other Pacific Islander. Thirty-one respondents (1%) did not report their race/ethnicity. Education was heterogenous: 1022 respondents (38%) had a high school education or less, 1254 respondents (47%) had some college or a 2-year degree, and 400 respondents (15%) had a 4-year degree or higher.

Compared with participants who viewed a map, not viewing a map was associated with greater knowledge about total cases (mean [SD] score, 0.60 [0.28] vs 0.55 [0.30]; difference, 0.05 [95% CI, 0.01 to 0.09]) (Table). However, knowledge about total cases was significantly better for maps showing total cases compared with maps showing per capita cases (mean [SD] score, 0.60 [0.30] vs 0.46 [0.28]; difference, 0.14 [95% CI, 0.11 to 0.17]).

**Table.** Planned Contrasts for Outcome Measures at Each Comparison Level

	Mean (SD) vs mean (SD)	Difference estimate (95% CIs)^a^
**Total cases knowledge**^**b**^		
Overall, mean (SD)	0.56 (0.30)	NA
No map vs map	0.60 (0.28) vs 0.55 (0.30)	0.05 (0.01 to 0.09)
Heat vs bubble	0.55 (0.30) vs 0.54 (0.30)	0.01 (−0.02 to 0.04)
State vs county	0.56 (0.30) vs 0.54 (0.30)	0.01 (−0.02 to 0.04)
Total vs per capita	0.60 (0.30) vs 0.46 (0.28)	0.14 (0.11 to 0.17)
**Cases per capita knowledge**^**c**^		
Overall	0.47 (0.25)	NA
No map vs map	0.44 (0.25) vs 0.47 (0.26)	−0.02 (−0.06 to 0.01)
Heat vs bubble	0.48 (0.26) vs 0.44 (0.24)	0.04 (0.01 to 0.06)
State vs county	0.49 (0.26) vs 0.45 (0.24)	0.04 (0.01 to 0.07)
Total vs per capita	0.42 (0.24) vs 0.56 (0.26)	−0.13 (−0.16 to −0.11)
**Individual risk perception**^**d**^		
Overall, mean (SD)	3.80 (1.11)	NA
No map vs map	3.83 (1.10) vs 3.79 (1.11)	0.04 (−0.12 to 0.20)
Heat vs bubble	3.81 (1.11) vs 3.75 (1.11)	0.06 (−0.07 to 0.18)
State vs county	3.78 (1.09) vs 3.80 (1.12)	−0.02 (−0.14 to 0.10)
Total vs per capita	3.81 (1.10) vs 3.75 (1.13)	0.06 (−0.06 to 0.19)
**Societal risk perception**^**e**^		
Overall, mean (SD)	3.98 (1.62)	NA
No map vs map	3.77 (1.60) vs 4.02 (1.62)	−0.25 (−0.48 to −0.02)
Heat vs bubble	3.99 (1.64) vs 4.06 (1.59)	−0.06 (−0.25 to 0.11)
State vs county	4.03 (1.63) vs 4.00 (1.61)	0.03 (−0.14 to 0.21)
Total vs per capita	4.03 (1.61) vs 3.99 (1.65)	0.04 (−0.15 to 0.22)
**Intentions to adhere to COVID-19 guidelines**^**f**^		
Overall, mean (SD)	86.33 (17.05)	NA
No map vs map	87.83 (16.93) vs 86.09 (17.06)	1.73 (−0.81 to 4.28)
Heat vs bubble	85.96 (17.24) vs 86.35 (16.69)	−0.38 (−2.41 to 1.65)
State vs county	85.84 (17.49) vs 86.34 (16.62)	−0.48 (−2.39 to 1.42)
Total vs per capita	86.05 (17.05) vs 86.17 (17.08)	−0.10 (−2.11 to 1.91)

Abbreviations: COVID-19, coronavirus disease 2019; NA, not applicable.

^a^ Outcomes of significance tests are implied by CIs that do not include zero.

^b^ Scores range from 0 to 1, with higher scores indicating greater knowledge about total numbers of confirmed COVID-19 cases.

^c^ Scores range from 0 to 1, with higher scores indicating greater knowledge about the number of confirmed COVID-19 cases per capita.

^d^ Scores range from 1 to 7 with higher scores indicating greater perceived susceptibility and severity of getting COVID-19.

^e^ Scores range from 1 to 7, with higher scores indicating more optimism that the pandemic would be much better in 2 weeks.

^f^ Scores range from 0 to 100, with higher scores indicating greater intent to adhere to the guidelines.

Viewing any map (vs no map) was not associated with knowledge about cases per capita. However, per capita knowledge was significantly better among respondents who viewed a heat map compared with those who viewed a bubble map (mean [SD] score, 0.48 [0.26] vs 0.44 [0.24]; difference, 0.04 [95% CI, 0.01 to 0.06]), the state-level map vs county-level map (mean [SD] score, 0.49 [0.26] vs 0.45 [0.24]; difference, 0.04 [95% CI, 0.01 to 0.07]), and the per capita map vs the total cases map (mean [SD] score, 0.42 [0.24] vs 0.56 [0.26]; difference, −0.13 [95% CI, −0.16 to −0.11]).

Respondents’ perception of their personal risk of getting COVID-19 was not associated with the presence or the type of map. Respondents who saw a map had lower societal risk perceptions, with more optimism that the pandemic would be better in 2 weeks, compared with those who did not see a map (mean [SD] score, 3.77 [1.60] vs 4.02 [1.62]; difference, −0.25 [95% CI, −0.48 to −0.02]). Overall, respondents reported high willingness to adhere to COVID-19 guidelines (mean [SD] score, 86.33 [17.05]), and scores were not significantly different by map provision or type.

## Discussion

The findings of this survey study suggest that simply providing maps with COVID-19 case information was not necessarily associated with improved public knowledge, risk perception, or reported intent to adhere to health guidelines.

Limitations of this study include reliance on self-report and potential limited participation from individuals without internet access and lower English proficiency.

Based on the findings of our survey study, we encourage map developers to be mindful of the potential influence of reporting strategies on public knowledge and perception of the pandemic. We suggest developers present cases per capita using state-level heat maps rather than county-level bubble maps, because the former may be associated with improving (or at least maintaining) public knowledge. Knowledge about strategies for effective communication of COVID-19 case information would benefit from research with other stakeholders, such as government officials or policy makers.
